# Classical Imaging with Undetected Photons

**DOI:** 10.1038/srep10329

**Published:** 2015-05-27

**Authors:** Jeffrey H. Shapiro, Dheera Venkatraman, Franco N. C. Wong

**Affiliations:** 1 Research Laboratory of Electronics, Massachusetts Institute of Technology, 77 Massachusetts Avenue, Cambridge, Massachusetts 02139, USA

## Abstract

Barreto Lemos *et al.* [*Nature* **512**, 409–412 (2014)] reported an experiment in which a non-degenerate parametric downconverter and a non-degenerate optical parametric amplifier—used as a wavelength-converting phase conjugator—were employed to image object transparencies in a manner akin to ghost imaging. Their experiment, however, relied on single-photon detection, rather than the photon-coincidence measurements employed in ghost imaging with a parametric downconverter source. More importantly, their system formed images despite the photons that passed through the object never being detected. Barreto Lemos *et al.* interpreted their experiment as a quantum imager, as assuredly it is, owing to its downconverter’s emitting entangled signal and idler beams. We show, however, that virtually all the features of their setup can be realized in a quantum-mimetic fashion using classical-state light, specifically a pair of bright pseudothermal beams possessing a phase-sensitive cross correlation. Owing to its much higher signal-to-noise ratio, our bright-source classical imager could greatly reduce image-acquisition time compared to that of Barreto Lemos *et al.*‘s quantum system, while retaining the latter’s ability to image with undetected photons.

Light is intrinsically quantum mechanical, and photodetection is a quantum measurement. Consequently, *all* imaging is really quantum mechanical. It has long been known, however, that the semiclassical theory of photodetection—in which light is a classical field and the discreteness of the electron charge results in photodetection shot noise—predicts measurement statistics identical to those obtained from quantum theory when the illumination is in a classical state, namely a Glauber coherent state or a classically-random mixture of such states. (See Ref. 1 for a review of quantum versus semiclassical photodetection.) Thus, because experiments whose quantitative behavior is correctly predicted by two disparate theories cannot distinguish between those two theories, it is entirely appropriate that the term quantum imaging be reserved for imagers whose quantitative understanding *requires* the use of quantum theory. (See Refs. 2, 3, 4 for how a debate on this point has been settled with regards to pseudothermal ghost imaging.)

Three-wave mixing in a second-order nonlinear material is the workhorse of nonclassical light-beam generation, with spontaneous parametric downconverters producing entangled signal and idler beams^5^, optical parametric amplifiers producing squeezed-vacuum states^6^, and optical parametric oscillators producing photon-twin beams^7^. It follows that imagers using any such sources will be quantum imagers, according to the criterion described in the preceding paragraph. Two such examples are the initial ghost-imaging experiment^8^, and quantum optical coherence tomography (Q-OCT)^9^,^10^. Both used continuous-wave (cw) spontaneous parametric downconversion (SPDC) sources, whose signal and idler outputs were taken to comprise streams of biphotons detectable by photon-coincidence counting. The joint state obtained from cw SPDC, however, is really a zero-mean Gaussian state that is completely characterized by the signal and idler’s nonzero correlations, viz., their phase-insensitive auto-correlations and their phase-sensitive cross correlation^11^. Signal-idler entanglement is then manifest by the phase-sensitive cross correlation’s exceeding the classical limit set by the auto-correlations. The biphoton limit ensues for the experiments reported in Refs. 8,10 because cw SPDC emits low-brightness outputs—average signal and idler photons/sec-Hz much less than 1—whose signal-idler photon pairs are easily resolved in time by high-speed photodetectors^11^.

Phase-sensitive cross correlations cannot be sensed in second-order interference. Hence the aforementioned experiments’ reliance on photon-coincidence counting, which is a fourth-order interference measurement able to sense phase-sensitive cross correlations^12^. More important, for our purpose, is the fact that *classical* light beams can have phase-sensitive cross correlations. Indeed, Ref. 13 showed theoretically, and Ref. 14 verified experimentally, that signal and idler beams in a zero-mean jointly Gaussian *classical* state—determined by their non-zero phase-insensitive auto-correlations and phase-sensitive cross-correlation—yielded ghost images with almost all of the properties of the quantum case.

Three-wave mixing can also be used to phase-conjugate a light beam that had only a phase-sensitive cross correlation with a companion beam. The resulting conjugate then has a phase-insensitive cross correlation with that companion, which can be sensed via second-order interference. This possibility was exploited, theoretically in Ref. 15 and experimentally in Ref. 16, to realize phase-conjugate optical coherence tomography, in which classical-state signal and idler beams—of the type mentioned in the preceding paragraph—yielded the axial resolution and dispersion immunity afforded by Q-OCT without the need for nonclassical light.

The upshot of our long introduction is simply this. Quantum imagers employing the entangled signal and idler beams obtained from SPDC often have quantum-mimetic relatives that use classical-state signal and idler beams to realize the essential performance characteristics of the original quantum systems. We show that the experiment of Barreto Lemos *et al.*^17^ is another such example. In particular, by replacing Barreto Lemos *et al.*‘s entangled-state source with a classical-state source whose signal and idler beams have a phase-sensitive cross correlation, we see that a wavelength-converting phase conjugator again allows imaging with undetected photons. Moreover, our system can employ high-brightness light, thus leading to a much higher signal-to-noise ratio than its quantum counterpart, and hence the possibility of greatly reduced image-acquisition time. Before proceeding to establish these results, two explanatory notes concerning “classical imaging with undetected photons” are warranted.

Whereas photons are energy quanta of electromagnetic waves, classical electromagnetic waves are not comprised of photons. So how can we do classical imaging with photons, much less with undetected photons? As explained in our opening paragraph, all imaging is quantum, but we have reserved the term quantum imaging for systems whose statistical descriptions require quantum photodetection theory. Imagers whose statistics can be correctly derived from semiclassical photodetection theory are classical-state (proper 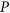-function) imagers, so a more precise description of our work would be to say we will analyze classical-state imaging with undetected photons. That clarification, however, still leaves us with the question of whether Barreto Lemos *et al.*‘s quantum imager or our classical-state imager form their images with *undetected* photons. Here the quantum explanation—applicable to both the quantum imager (with its nonclassical light source) and the classical-state imager (the former’s quantum-mimetic counterpart)—is as follows. Spontaneous parametric downconversion in the wavelength-converting phase conjugator fissions a small fraction of the pump photons into signal-idler photon pairs. But the presence of incoming idler photons causes that three-wave mixer to emit additional signal-idler photon pairs. Thus, for both imagers considered below, the photons that interacted with the object being imaged are indeed *not* the photons that are detected.

## Results

**Figure 1:**
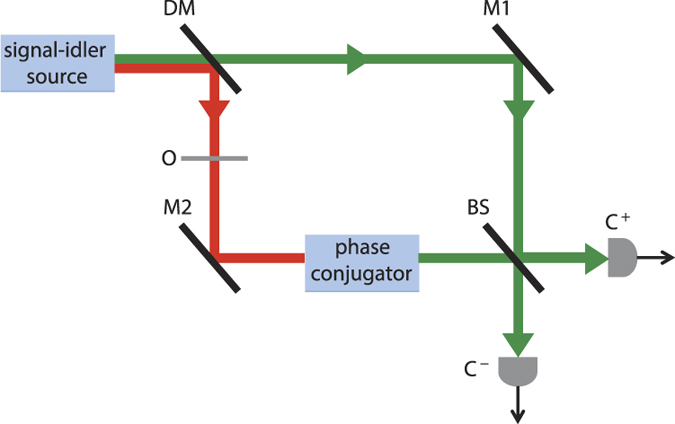
Setup for quantum and classical imaging with undetected photons. DM dichroic mirror; M1, M2 mirrors; O object transparency; BS 50–50 beam splitter; C^+^, C^−^ cameras.

The setup we shall consider, which mimics that of Ref. 17, is shown in Fig. 1. Here, a signal-idler source produces a wavelength *λ*_*s*_ signal beam (green beam in Fig. 1) and wavelength *λ*_*i*_ idler beam (red beam in Fig. 1) that are separated by a dichroic mirror. The idler beam then propagates through an object transparency and a wavelength-converting phase conjugator whose output at wavelength *λ*_*s*_ is mixed with the signal on a 50–50 beam splitter. The beam splitter’s outputs are detected by cameras whose outputs, we will show, contain positive and negative images of the object transparency, just as reported by Barreto Lemos *et al.*^17^ for their quantum imager. Lenses, not shown in Fig. 1, image the source’s idler-beam output onto the object transparency and the idler light transmitted through that transparency onto the phase conjugator. Other lenses, also omitted from Fig. 1, image the source’s signal-beam output and the signal-wavelength output from the phase conjugator onto the cameras, completing an equal path-length interferometer.

For a unified treatment of the quantum and quantum-mimetic versions of the Fig. 1 setup, we shall employ quantum analysis for both. Quantitatively identical results are obtained for the quantum-mimetic case when: (1) we use classical wave propagation for the object-transparency and beam-splitter interactions; (2) we use the classical-noise model for the action of the wavelength-converting phase conjugator; and (3) we use the semiclassical (shot-noise plus illumination excess-noise) theory of photodetection. For simplicity, in what follows, we shall discretize in both space and time, i.e., we will take the source’s signal and idler outputs to be a collection of modes with photon-annihilation operators 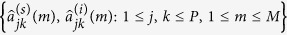 for a *P *× *P* array of pixels, indexed by (*j,k*), and a sequence of 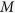 pulses, indexed by 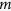. These modes will be assumed to be in a zero-mean jointly-Gaussian state whose non-zero correlation functions are 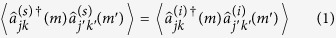


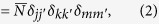
 and 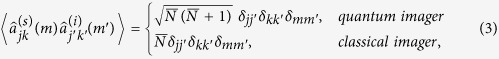 where *δ*_*ab*_ is the Kronecker delta function. Equations (1, 2, 3) imply pixel-to-pixel and pulse-to-pulse statistical independence, with the quantum imager having the maximum quantum-mechanical phase-sensitive cross correlation given its outputs’ average of 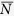 photons per mode, and the classical imager having its maximum classical phase-sensitive cross correlation under this same constraint. Note, however, that the SPDC source used to produce the quantum imager’s signal and idler beams operates at low brightness, 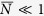. On the other hand, the classical imager’s signal and idler beams—which can be obtained by using spatial light modulators to apply phase-conjugate pseudorandom modulations to wavelength *λ*_*s*_ and *λ*_*i*_ laser beams—can have high brightness, 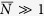. Thus, to disambiguate these two cases in all that follows, we shall use 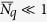 and 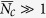 to denote the average per-mode photon numbers for the quantum and classical imagers, respectively.

Regardless of whether the source is quantum or classical, the annihilation operators for the wavelength-*λ*_*i*_ modes emerging from the object transparency are 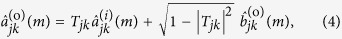 where *T*_*jk*_ is the object’s complex-valued field transmission at pixel (*j,k*), and the 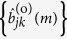 are the annihilation operators for a collection of vacuum-state auxiliary modes associated with transmission loss through the object. The annihilation operators for the wavelength-*λ*_*s*_ modes emerging from the phase conjugator are then 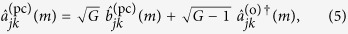 where *G*>1 is the conjugator’s gain, assumed to be real valued, and the 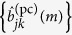 are the annihilation operators for the collection of vacuum-state wavelength-*λ*_*s*_ modes at the conjugator’s input. Here, because we have assumed that the gain value is the *same* for all pixels, it is crucial that the conjugator be a spatially-broadband device. As such it is very likely to have 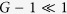, e.g., it could be an SPDC system into which the 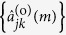 modes are injected. Indeed, if the conjugator uses the same crystal and pump power as the quantum imager’s signal-idler source, then 
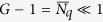
 will prevail, even for the classical imager. In what follows we will make that assumption. Note that the classical model for the conjugator, when the source emits classical-state light, is then 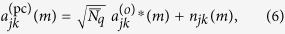 where classical complex amplitudes 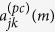 and 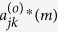 have replaced the annihilation and creation operators 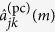 and 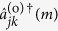, and 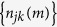 is a set of independent, identically-distributed, zero-mean, circulo-complex Gaussian random variables characterized by 
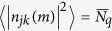
.

The cameras record 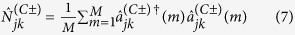 via photon counting (for the low-brightness quantum system) or shot-noise-limited direct detection (for the high-brightness classical system), where 
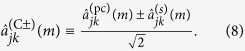


To compare the images that the quantum and classical systems provide, we first evaluate their ensemble-average behaviors. It is easy to show, for the states we have assumed, that 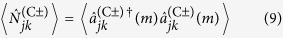


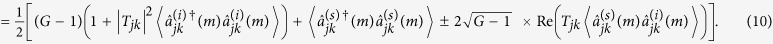


Specializing equation (10) to the quantum (subscript *q*) and classical (subscript *c*) cases gives 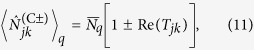 and 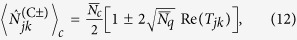 where we have used 
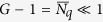
 in both cases and 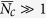 for the classical case. Equation (11) agrees with the theory from Ref. 17: cameras C^+^ and C^−^ produce positive and negative images, respectively, of Re(*T*_*jk*_) with 100% fringe visibilities when 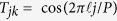 for 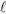 a nonzero integer. In comparison with Barreto Lemos *et al.*‘s quantum imager, equation (12) shows that our classical imager produces positive and negative images of Re(*T*_*jk*_) with much weaker, 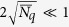, fringe visibilities for 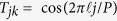.

We define the positive and negative images’ signal-to-noise ratios (SNRs), for *ν*=*q*,*c*, by 
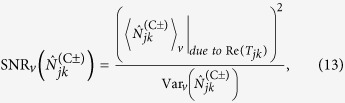
 where the subscript in the numerator is used to indicate that we only include the object-related portion of the average images, not the object-independent background terms that appear in equations (11) and (12), in assessing their strengths. Now, because the 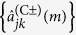 are in thermal states for both the quantum and classical cases, we immediately find that 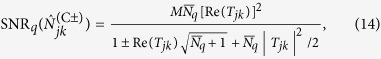 and 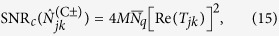 for 
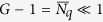
 and 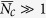. Thus the positive and negative quantum images have different SNRs, whereas the positive and negative classical images have identical SNRs. More importantly, 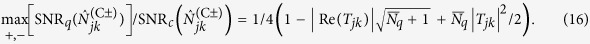


For *T*_*jk*_=±1, this SNR ratio approaches 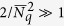, making it seem that the classical imager is vastly inferior to the quantum imager. For 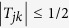, however, this SNR ratio lies between 1/4 and 1/2, indicating that the quantum imager is slightly worse than the classical imager for low transmissivity objects. Such, however, is *not* the case, as we now demonstrate by examining the behavior of the difference images, 
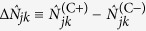
, for the quantum and classical systems.

The difference-image mean values, which follow immediately from equations (11) and (12), are both proportional to Re(*T*_*jk*_), so we define their SNRs by 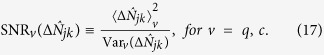


These SNRs are easily found using 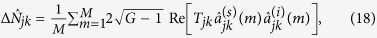 and 
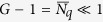
, 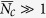. The results, 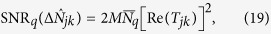 and 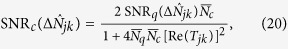 clearly indicate the SNR advantage afforded by the classical imager’s use of a high-brightness source. That advantage can translate into a much shorter image-acquisition time than that of the quantum imager, with its low-brightness SPDC source. In particular, for all pixels with |Re(*T*_*jk*_)|≠0, we have that 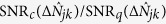 increases monotonically with increasing 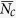. For 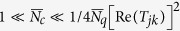 this SNR advantage equals 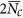, and it saturates at 
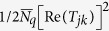
 as 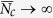.

The huge improvement in the classical imager’s SNR that accrues from subtracting its negative image from its positive image is easily explained from semiclassical theory. In particular, because 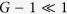 and 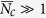, the noise in the classical system’s positive and negative images is overwhelmingly variance-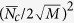 excess noise from the signal light illuminating the two detectors. For the classical system’s difference image, however, the complete correlation between the signal-beam excess noises impinging on the two detectors greatly reduces their impact on the difference image. Indeed, when the signal-wavelength output from the phase conjugator is blocked, the difference image’s variance is reduced to 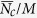, the sum of the two detectors’ shot noises. However, when both beams interfere at the 50–50 beam splitter, there is additional noise in the difference image. Its origin is easily identified if we interpret the difference image as arising from a balanced-homodyne measurement, in which the signal beam in the classical imager acts as a strong local oscillator (LO) that beats with the weak signal-wavelength output from the phase conjugator. The extra noise just alluded to is then the LO fluctuations’ random modulation of the beat between the two fields^18^. Thus, in equation (20), the first term in the denominator comes from the shot noise and the second term in the denominator, which sets the 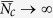 upper bound on the classical imager’s SNR advantage, comes from the modulation noise.

## Discussion

SPDC sources can be used to produce a wide variety of entangled and even hyper-entangled states. When such a source’s polarization-entangled outputs are used to perform qubit teleportation^19^, or its quadrature-entangled outputs are used to perform continuous-variable teleportation^20^, the results are fundamentally quantum mechanical, in that classical resources cannot approach the fidelities achievable with entanglement-based teleportation. A somewhat different situation transpires when SPDC sources are used in imagers. Here, previous work on ghost imaging^13^,^14^ and optical coherence tomography^15^,^16^ has shown that almost all of the features of SPDC-based systems can be realized with quantum-mimetic counterparts that use classical-state light, namely pseudothermal signal and idler beams with a phase-sensitive cross correlation. We have demonstrated the same to be true for Barreto Lemos *et al.*‘s quantum imaging with undetected photons^17^. In particular, we have analyzed a classical-state system capable of imaging an object transparency at wavelength *λ*_*i*_ by sensing only wavelength-*λ*_*s*_ photons, a wavelength for which the object may be opaque. Moreover, our classical-state system can use a bright source that results in its having a much higher SNR than that of its quantum counterpart, owing to the latter’s use of a low-brightness source. Thus classical imaging with undetected photons can have a much shorter image-acquisition time than quantum imaging with undetected photons.

**Figure 2:**
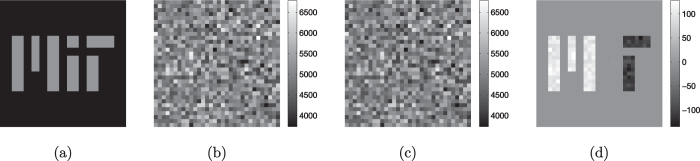
Object transparency and simulated classical-state images for 32×32 pixels: (**a**) |*T*_*jk*_|^2^ for the MIT logo transparency with “M” having *T*_*jk*_=1/2, “I” having *T*_*jk*_=*e*^*iπ*/2^/2, “T” having *T*_*jk*_=−1/2, and *T*_*jk*_=0 for all other pixels; (**b**) positive image; (**c**) negative image; and (**d**) difference image. The images in (**b**–**d**) and were generated using 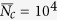, 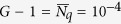, and *M*=100.

To illustrate the behavior of our quantum-mimetic system, we have performed computer simulations of the positive, negative and difference images, 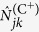, 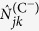, and 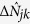. The object transparency, whose |*T*_*jk*_|^2^ is shown in Fig. 2(a), was a 32×32 MIT logo in which the “M” had field transmission *T*_*jk*_=1/2, the “I” had field transmission *T*_*jk*_=*e*^*iπ*/2^/2, the “T” had field transmission *T*_*jk*_=−1/2, and all other pixels had *T*_*jk*_=0. Figure 2(b–d) are the resulting positive, negative, and difference images, respectively, when 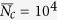, 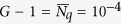, and *M*=100. These images show the characteristics expected from our analysis: (1) low contrast, low SNR in the positive and negative images, but high contrast and high SNR in the difference image; and (2) difference image with positive values for its “M” pixels, near-zero values for its “I” pixels, and negative values for its “T” pixels. Note that a quantum difference image of this transparency—taken with the same 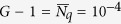 and *M* = 100 values—would have an SNR 40 dB lower than that of the classical difference image.

At this juncture some final overarching comments are in order. The work of Barreto Lemos *et al.*^17^ is, in essence, a spatially-broadband version of the quantum interference experiment of Zou, Wang, and Mandel^21^. Given that we have shown the former has a quantum-mimetic (classical-state) counterpart which retains that system’s essential imaging characteristics, it behooves us to comment on whether a similar situation prevails with respect to the latter. Indeed it does. The Zou, Wang, and Mandel experiment is assuredly quantum: it employs nonclassical light and hence requires quantum photodetection theory for its analysis, which those authors accomplish via the biphoton approximation for the post-selected outputs from an SPDC source. That said, however, our Gaussian-state treatment of SPDC is more rigorous; for example, it includes the multiple-pair emissions that account for the accidental coincidences seen in Hong-Ou-Mandel interferometry even when detector dark-counts are negligible^1^,^12^. Using Gaussian-state analysis, the quantum versus quantum-mimetic behavior for Ref. 21‘s experiment is explained by the single-pixel, *M*-pulse, object transmissivity *T*=*e*^*iφ*^ version of our theory, where *φ* is a controllable phase shift. In particular, from equations (11) and (12) we see that both the Zou, Wang, and Mandel experiment and our classical-state version thereof exhibit sinusoidal fringes as *φ* is varied, but the nonclassical source provides 100% fringe visibility, while the classical-state source yields very low fringe visibility.

An additional point, which emerges from our Gaussian-state analyses of^17^,^21^, is that both rely on *stimulated*, rather than spontaneous, emissions from the wavelength-converting phase conjugator in Fig. 1. This conclusion follows from equation (5) and 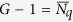, which readily yield 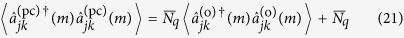 for the average photon number of the conjugator’s *m*th signal-pulse output at pixel (*j,k*). Here, the first term on the right represents signal photons whose emissions were stimulated by the presence of idler photons at the conjugator’s input, while the second term on the right represents signal photons whose emissions occurred spontaneously. That stimulated emissions are responsible for the quantum and classical-state images we found earlier is then obvious from the resulting nonzero phase-insensitive cross correlation between the two signal-wavelength beams arriving at the beam splitter in Fig. 1, i.e., 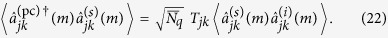


This cross correlation, which must be nonzero for the cameras in Fig. 1 to record an image, vanishes in the absence of stimulated signal emissions from the conjugator.

Equation (22) brings us to our paper’s final point. Just how essential is a wavelength-converting phase conjugator to quantum and classical imaging with undetected photons? For the quantum case the answer is clear. The phase-sensitive cross correlation between the signal and idler beams produced by cw SPDC cannot be sensed in second-order interference, but the phase-conjugating nature of Fig. 1’s wavelength converter transforms phase-sensitive cross correlation into phase-insensitive cross correlation, which *can* be sensed in second-order interference. The same necessity for phase conjugation arises if we replace the SPDC source with a classical-state source possessing phase-sensitive (but not phase-insensitive) cross correlation between its signal and idler outputs. But why must our source only produce a phase-sensitive cross correlation between its two output beams in order to image with undetected photons? The answer is that it need not. Suppose that the source in Fig. 1 produces a wavelength *λ*_*s*_ signal beam and a wavelength *λ*_*i*_ idler beam whose modal annihilation operators, 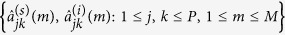, are in a zero-mean, jointly-Gaussian state with non-zero correlation functions given by equations (1), (2), and 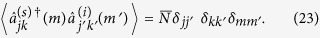


Despite this phase-insensitive cross-correlation function’s being at the limit set by quantum physics, the joint state of the signal and idler is classical^22^. Now assume that *λ*_*s*_ < *λ*_*i*_, and that the wavelength-converting phase conjugator in Fig. 1 is replaced with a spatially-broadband upconverter whose output modes at wavelength *λ*_*s*_ are given by 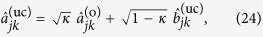 where 0 < *κ* < 1 is the conversion efficiency and the 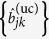 are in their vacuum states. It is easily shown, by paralleling the derivation that we presented earlier for a phase-sensitive cross correlation, that cameras C^+^ and C^−^ will record background-embedded positive and negative images, respectively, of Re(*T*_*jk*_), even when the object is opaque at the signal wavelength. In fact, having introduced the use of a spatially-broadband upconverter, we can do classical imaging with undetected photons in an even simpler manner: use laser light at wavelength *λ*_*i*_ to uniformly illuminate an object transparency that is opaque at wavelength *λ*_*s*_<*λ*_*i*_. Then, use a spatially-broadband upconverter—described by equation (24)—to obtain an image of |*T*_*jk*_|^2^ by casting the wavelength *λ*_*s*_ field emerging from this three-wave mixer on a camera.

## Additional Information

**How to cite this article**: Shapiro, J. H. *et al.* Classical Imaging with Undetected Photons. *Sci. Rep.* **5**, 10329; doi: 10.1038/srep10329 (2015).
